# Sicherheit von Blut und Blutprodukten: Testmethoden zum Nachweis der Hepatitisviren B, C und E

**DOI:** 10.1007/s00103-021-03480-0

**Published:** 2022-01-13

**Authors:** Johanna Mitterreiter, Heinrich Scheiblauer, Sarah Fiedler, Julia Kreß

**Affiliations:** 1 Fachgebiet Molekulare Virologie, Paul-Ehrlich-Institut, Paul-Ehrlich-Straße 51–59, 63225 Langen, Deutschland; 2 Prüflabor für In-vitro-Diagnostika, Paul-Ehrlich-Institut, Langen, Deutschland; 3 Abteilung Sicherheit von Arzneimitteln und Medizinprodukten, Paul-Ehrlich-Institut, Langen, Deutschland

**Keywords:** Hepatitisviren, Blutspenderscreening, NAT, Antigentest, Blutsicherheit, Hepatitis virus, Blood screening, NAT, Antigen testing, Blood safety

## Abstract

Infektionen mit den Hepatitisviren B, C und E (HBV, HCV, HEV) sind über das Blut übertragbar und können schwere akute und chronische Leberentzündungen hervorrufen. Um die Sicherheit von Spenderblut zu gewährleisten und Empfänger vor Virusübertragungen zu schützen, werden Blutkonserven in Deutschland mit Nukleinsäureamplifikationstechniken (NAT) auf Virusgenom sowie mit serologischen Methoden auf virale Antigene und humane Antikörper getestet. In diesem Beitrag werden die entsprechenden Regularien zur Sicherheit von Blut und Blutprodukten in Deutschland sowie die verschiedenen Screeningmethoden beschrieben. Die Sicherheit der Blutprodukte wird bewertet.

Beim Spenderscreening angewandte NAT-Methoden basieren auf Technologien der Polymerasekettenreaktion (PCR) oder auf isothermen Verfahren, wie der *Transcription-mediated Amplification* (TMA), welche einen hochempfindlichen Nachweis einer Virusinfektion in Spenderblut ermöglichen und so zu einer Verkürzung der diagnostischen Fensterperiode beitragen. Bereits seit den 1970er-Jahren wird zur Erkennung einer möglichen HBV-Infektion ein Screening des viralen Oberflächenproteins (HBsAg) gefordert. Die verpflichtende Einführung der Testung auf HCV-spezifische Antikörper 1992, der HCV-NAT-Testung 1999 und des Anti-HBc-Spenderscreenings 2006 sowie die nichtverpflichtende, von den meisten Blutspendeeinrichtungen freiwillig durchgeführte HBV-NAT-Testung haben die Sicherheit von Blutprodukten deutlich verbessert und Übertragungsfälle auf seltene Einzelfälle in der frühen diagnostischen Fensterperiode reduziert. Der Erfolg der 2020 in Deutschland eingeführten HEV-NAT-Testung von Spenderblut wird sich in den kommenden Jahren bemessen lassen. Neben der Spendertestung ergänzen Maßnahmen zur Spenderauswahl und Pathogeninaktivierung das Sicherheitssystem für Blutspenden in Deutschland.

## Abstract (en)

Infections with hepatitis B, C, and E virus (HBV, HCV, and HEV) can be transmitted via blood and cause severe acute or chronic liver infections. To ensure the safety of blood donations and protect recipients from virus transmissions, blood donations in Germany are tested for viral genomes using nucleic acid amplification techniques (NATs) as well as for viral antigens and antibodies by serological testing. This article describes the relevant regulations on the safety of blood and blood products in Germany and the various screening methods. The safety of blood products is assessed.

Currently used NAT methods for detection of hepatitis viruses are based either on polymerase chain reaction (PCR) or isothermal methods such as transcription-mediated amplification (TMA), which enable a highly sensitive detection of viral infections and thereby contribute to the reduction of the diagnostic window. Antigen tests for the detection of viral surface protein of hepatitis B virus in blood donations were introduced in the 1970s in order to prevent potential transmissions. Since the introduction of mandatory testing for HCV-specific antibodies in 1992, HCV NAT testing in 1999, anti-HBc antibody testing in 2006, and the non-mandatory HBV NAT, which is voluntarily performed by most of the blood establishments, blood safety has increased tremendously. Only a few isolated cases of transfusion-transmitted infections in the early window period have been reported since. The success of the recent introduction of mandatory HEV NAT testing in 2020 will have to be assessed in the upcoming years. Besides blood donor screening, the system for blood safety in Germany is supplemented by additional measures for donor selection and pathogen inactivation.

## Hintergrund

Weltweit sterben jährlich noch weit über 1 Mio. Menschen an den Folgen einer viralen Hepatitis. Hepatitiden können von zahlreichen Viren hervorgerufen werden, der Großteil der viralen Hepatitiden geht jedoch auf primär hepatotrope Erreger zurück: die Hepatitisviren A bis E (HAV–HEV). Hierbei sind vor allem das HBV und das HCV mit weltweit etwa 250 Mio. chronisch HBV-infizierten und 70 Mio. chronisch HCV-infizierten Menschen weit verbreitet [1]. Da diese beiden Virusarten über Blut übertragen werden können, lag auf ihnen in den letzten 20 Jahren ein Hauptaugenmerk der Transfusionsmedizin bei der Regulierung von Blut und Blutprodukten.

Nachdem es in den 1980er- und 1990er-Jahren zu vermehrten Übertragungen des Humanen Immundefizienz-Virus (HIV) durch Spenderblut gekommen war, wurde das Testregime aller Blutspenden systematisch erweitert, sodass es mittlerweile neben HBV und dem bakteriellen Syphiliserreger auch HIV und HCV abdeckt. Ein stetiger Zuwachs an gemeldeten HEV-Infektionen in den letzten 10 Jahren und internationale Berichte über chronische HEV-Fälle durch Bluttransfusionen führten zudem ab dem 01.01.2020 zur Einführung des zusätzlichen Screenings von HEV in Blutspenden [2–4]. Im Gegensatz dazu unterstehen Blut und Blutprodukte in Deutschland keiner systematischen Testung auf HAV oder HDV. Während HAV hauptsächlich über die fäkal-orale Route übertragen wird, ist HDV helfervirusabhängig und benötigt das Oberflächenprotein von HBV (HBsAg) als Hüllprotein, wodurch es zusammen mit dem Screening auf HBsAg abgedeckt wird.

Dieser Artikel beschreibt die aktuell in Deutschland geltenden Regularien zur Sicherheit von Blut und Blutprodukten im Hinblick auf transfusionsbedingte HBV-, HCV- und HEV-Übertragungen sowie die dafür eingesetzten Screeningmethoden. Zudem wird der Erfolg dieser Maßnahmen und somit die Sicherheit von in Deutschland verwendeten Blutprodukten bewertet.

## Regularien in Deutschland

Europaweit regeln die Richtlinie 2002/98/EG [5] und die nachfolgenden technischen Richtlinien des Europäischen Parlaments und des Rates die grundlegenden Anforderungen für Qualitäts- und Sicherheitsstandards von menschlichem Blut und Blutbestandteilen für die Transfusionsmedizin. In Deutschland erfolgt die Umsetzung dieser Direktiven durch das Transfusionsgesetz (TFG; [6]), das Arzneimittelgesetz (AMG; [7]) und nachfolgende Verordnungen. Detaillierte Anforderungen an das Blutspendewesen in Deutschland sind in der Hämotherapie-Richtlinie [8] der Bundesärztekammer festgelegt. Der Arbeitskreis Blut, ein Expertengremium angesiedelt am Robert Koch-Institut, berät zudem die Bundes- und Landesbehörden in Sachen Blutsicherheit und veröffentlicht seine Beschlüsse als sogenannte Voten. Darüber hinaus kann das Paul-Ehrlich-Institut (PEI) als zuständige Bundesoberbehörde bei neuen wissenschaftlichen Erkenntnissen in einem Stufenplanverfahren spezielle Maßnahmen zur Erhöhung der Sicherheit von Blutprodukten vorschreiben.

In einem derartigen Stufenplanverfahren führt das PEI je nach Wissensstand zunächst einen Informationsaustausch mit den betroffenen Blutspendeeinrichtungen durch, um den Grad der Gefährdung zu erfassen und mögliche Maßnahmen der Risikominimierung zu diskutieren. Das PEI kann auch direkt zu einer beabsichtigten Auflage anhören und so Informationen zur Machbarkeit erhalten, die dann im finalen Stufenplanbescheid berücksichtigt werden können. Bei Dringlichkeit kann das PEI auch ohne Anhörung gemäß § 28 AMG die Zulassung mit Auflagen verbinden, um die Sicherheit aufrechtzuerhalten. Bisher veröffentlichte Stufenplanbescheide des PEI betrafen Auflagen zum Spenderscreening, also zur Testung auf Antigen-Antikörper- bzw. Erregerbestandteile, Auflagen zur Spenderauswahl oder auch Auflagen, die sich auf Herstellungsschritte bezogen.

Die aktuell gültigen Anforderungen an das Screening von Blutspenden auf Virusinfektionen sind in Tab. 1 zusammengefasst. Allgemein wird zwischen serologischen Screeningmethoden auf Antigene bzw. Antikörper und Nukleinsäureamplifikationstechniken (NAT) zur Testung auf Virusgenom unterschieden. Während für HCV sowohl die Testung der Antikörper als auch eine HCV-NAT vorgeschrieben sind, ist für das HBV-Screening eine serologische Testung auf Anti-HBc-Antikörper und das HBs-Antigen ausreichend [9–11]. In den meisten Blutspendeeinrichtungen wird außerdem eine NAT-Testung auf HBV-DNA routinemäßig auf freiwilliger Basis durchgeführt. Für HEV wurde ab 2020 die HEV-NAT verpflichtend für das Screening von Blutspenden eingeführt [12].

**Table 1:** 

Marker	Serologie	NAT
HBV	Anti-HBc ^a^, HBsAg	–
HCV	Anti-HCV	HCV
HEV	–	HEV
HIV-1/2	Anti-HIV-1/2	HIV-1/2
WNV ^b^	–	WNV
Syphilis	Anti-Treponema pallidum	–

^a^ Anti-HBc-reaktive und HBsAg-negative Blutspenden dürfen freigegeben werden, wenn 2 weitere, vom initialen verwendeten Screeningtest verschiedene Anti-HBc-Testsysteme (oder ein Anti-HBc-Bestätigungstest) und eine Testung auf HBV-Genome mittels NAT (Mindestsensitivität ≤ 5 IE/mL) negative Testergebnisse ergeben [10, 38]

^b^ *WNV* West-Nil-Virus. Von der saisonalen (1.06.–30.11.), 4‑wöchigen Spenderrückstellung kann durch ein WNV-NAT-Screening abgewichen werden

*NAT* Nukleinsäureamplifikationstechniken, *HBV* Hepatitis-B-Virus, *HCV* Hepatitis-C-Virus, *HEV* Hepatitis-E-Virus, *HIV* Humanes Immundefizienz-Virus, *WNV* West-Nil-Virus, *HBc* Hepatitis-B-Virus Oberflächenprotein, *HBsAg* Hepatitis-B-Virus Core-Antigen

Blutspendeeinrichtungen und pharmazeutische Unternehmer müssen alle für das Blutspenderscreening eingesetzten Prüfverfahren für die in Tab. 1 gelisteten Infektionsmarker beim PEI anzeigen. Diese werden dann auf ihre Eignung überprüft und bei Erfüllung aller Anforderungen in eine Datenbank „Spendertestung“ aufgenommen [11]. Tab. 2 enthält eine Übersicht der 2021 in Deutschland gemeldeten Screeningtests für HBV, HCV und HEV. Neben kommerziell erhältlichen Tests, die durch ein Zertifizierungsverfahren (CE) speziell für das Blutspenderscreening zugelassen sind, dürfen auch solche kommerziellen Tests verwendet werden, die nicht primär für das Blutscreening zugelassen sind (sog. Off Label Use). Auch eigens etablierte „In-Haus“-Methoden dürfen verwendet werden – sofern sie den vom PEI festgelegten Anforderungen entsprechen und die notwendigen Validierungen vorgenommen wurden.

**Table 2:** 

Marker	Testname	Hersteller	Testprinzip	Messprinzip
HBV DNA	cobas MPX (6800/8800 System)	Roche Diagnostics	Screening (multiplex)	PCR
HBV DNA	cobas TaqScreen MPX Test, v2.0	Roche Diagnostics	Screening (multiplex)	PCR
HBV DNA	Procleix Ultrio Elite Assay	Grifols Diagnostic Solutions Inc	Screening (multiplex)	TMA
HBV DNA	Procleix Ultrio Plus Assay	Grifols Diagnostic Solutions Inc	Screening (multiplex)	TMA
HBV DNA	Procleix Ultrio Assay	Grifols Diagnostic Solutions Inc	Screening (multiplex)	TMA
HBV DNA	Virus Screening PCR Kit, v1.3	GFE Blut mbH	Screening (multiplex)	PCR
HCV RNA	cobas MPX (6800/8800 System)	Roche Diagnostics	Screening (multiplex)	PCR
HCV RNA	cobas TaqScreen MPX Test, v2.0	Roche Diagnostics	Screening (multiplex)	PCR
HCV RNA	Procleix Ultrio Elite Assay	Grifols Diagnostic Solutions Inc	Screening (multiplex)	TMA
HCV RNA	Procleix Ultrio Plus Assay	Grifols Diagnostic Solutions Inc	Screening (multiplex)	TMA
HCV RNA	Procleix Ultrio Assay	Grifols Diagnostic Solutions Inc	Screening (multiplex)	TMA
HCV RNA	Virus Screening PCR Kit, v1.3	GFE Blut mbH	Screening (multiplex)	PCR
HEV RNA	cobas HEV (6800/8800 System)	Roche Diagnostics	Screening	PCR
HEV RNA	Procleix HEV Assay	Grifols Diagnostic Solutions Inc	Screening	TMA
Anti-HBc	Alinity i Anti-HBc II Reagenz	Abbott GmbH	Indirekt (Anti-Human-IgG, -IgM)	CMIA
Anti-HBc	Alinity s Anti-HBc Reagenz	Abbott GmbH	Indirekt (Anti-Human-IgG, -IgM)	CMIA
Anti-HBc	Architect Anti-HBc II Reagenz	Abbott GmbH	Indirekt (Anti-Human-IgG, -IgM)	CMIA
Anti-HBc	Prism HBcore Reagenz	Abbott GmbH	Kompetitiv	CLIA
Anti-HBc	Access HBc Ab	Beckman Coulter	Indirekt (Protein A)	CLIA
Anti-HBc	Monolisa Anti-HBc Plus	Bio-Rad	Indirekt (Anti-Human-IgG, -IgM)	ELISA
Anti-HBc	Liaison Anti-HBc	DiaSorin S.p.A.	Kompetitiv	CLIA
Anti-HBc	Murex anti-HBc (total)	DiaSorin S.p.A. – UK Branch	Kompetitiv	ELISA
Anti-HBc	Anti-HBc	Roche Diagnostics	Kompetitiv	ECLIA
Anti-HBc	Elecsys Anti-HBc II	Roche Diagnostics	Kompetitiv	ECLIA
Anti-HBc	Advia Centaur Anti-HBc Total (HBcT)	Siemens Healthcare Diagnostics	Sandwich	CLIA
Anti-HBc	Advia Centaur HBc Total 2 (HBcT2)	Siemens Healthcare Diagnostics	Sandwich	CLIA
Anti-HBc	Atellica IM Anti Hepatitis B core Total (HBcT)	Siemens Healthcare Diagnostics	Sandwich	CLIA
Anti-HBc	Atellica IM HBc Total 2 (HBcT2)	Siemens Healthcare Diagnostics	Sandwich	CLIA
HBsAg	Alinity i HBsAg Qualitative II Reagenz	Abbott GmbH	Sandwich	CMIA
HBsAg	Alinity i HBsAg Reagenz	Abbott GmbH	Sandwich (quantitativ)	CMIA
HBsAg	Alinity s HBsAg Reagenz	Abbott GmbH	Sandwich	CMIA
HBsAg	Architect HBsAg Qualitative II Reagenz	Abbott GmbH	Sandwich	CMIA
HBsAg	Prism HBsAg Reagenz	Abbott GmbH	Sandwich	CLIA
HBsAg	Access HBsAg	Beckman Coulter	Sandwich	CLIA
HBsAg	Liaison XL murex HBsAg Quant	DiaSorin S.p.A.	Sandwich (quantitativ)	CLIA
HBsAg	Elecsys HBsAg II	Roche Diagnostics	Sandwich	ECLIA
HBsAg	HBsAg II	Roche Diagnostics	Sandwich	ECLIA
HBsAg	Advia Centaur HBsAg II (HBsII)	Siemens Healthcare Diagnostics	Sandwich	CLIA
HBsAg	Atellica IM Hepatitis B surface Antigen II (HBsII)	Siemens Healthcare Diagnostics	Sandwich	CLIA
Anti-HCV	Alinity i Anti-HCV Reagenz	Abbott GmbH	Indirekt (Anti-Human-IgG/IgM)	CMIA
Anti-HCV	Alinity s Anti-HCV Reagenz	Abbott GmbH	Sandwich	CMIA
Anti-HCV	Architect Anti-HCV Reagenz	Abbott GmbH	Indirekt (Anti-Human-IgG/IgM)	CMIA
Anti-HCV	Prism HCV Reagenz	Abbott GmbH	Indirekt (Anti-Human-IgG)	CLIA
Anti-HCV	Liaison XL murex HCV Ab	DiaSorin S.p.A.	Indirekt (Anti-Human-IgG)	CLIA
Anti-HCV	HCV 3.0 ELISA Test System with Enhanced SAVe	Ortho – Clinical Diagnostics	Indirekt (Anti-Human-IgG)	ELISA
Anti-HCV	Anti-HCV II	Roche Diagnostics	Sandwich	ECLIA
Anti-HCV	Elecsys Anti-HCV II	Roche Diagnostics	Sandwich	ECLIA
Anti-HCV	Advia Centaur Anti-HCV	Siemens Healthcare Diagnostics	Indirekt (Anti-Human-IgG)	CLIA
Anti-HCV	Atellica IM Hepatitis C (aHCV)	Siemens Healthcare Diagnostics	Indirekt (Anti-Human-IgG)	CLIA
HCV Ag/Ab	Murex HCV Ag/Ab Combination	DiaSorin S.p.A. – UK Branch	Sandwich	ELISA

*PCR* Polymerasekettenreaktion, *TMA* Transcription-mediated Amplification, *CMIA* Chemiluminescent Microparticle Immunoassay, *CLIA* Chemolumineszenz-Immunoassay, *ELISA* Enzyme-linked Immunosorbent Assay, *ECLIA* Elektrochemilumineszenz-Immunoassay

## Prävalenz, Übertragung, Infektionsverläufe und Testung bei HBV, HCV und HEV

Taxonomisch betrachtet handelt es sich bei Hepatitisviren um sehr unterschiedliche Viren, die ihren Namen nur aufgrund der gleichen primär induzierten Erkrankung teilen.

### HBV

HBV ist das am weitesten verbreitete Hepatitisvirus mit weltweit etwa 250 Mio. chronisch Infizierten und geschätzten 887.000 Todesfällen im Jahr 2015 [1]. In Deutschland wurden 2019 knapp 9000 HBV-Fälle gemeldet. Die Übertragung erfolgt durch Kontakt mit Blut oder anderen Körperflüssigkeiten [13]. Der primäre Übertragungsweg ist in Deutschland der sexuelle Kontakt. Im Gegensatz zu HCV und HEV gibt es eine effektive Impfung gegen HBV. Diese ist seit 1995 Bestandteil der empfohlenen Standardimpfungen für Säuglinge, Kinder, Jugendliche und Erwachsene. HBV gehört zur Familie der *Hepadnaviridae* und hat ein zirkuläres, teilweise doppelsträngiges DNA-Genom. Dieses wird von einem Kapsid aus Core-Antigen (HBcAg) umschlossen, welches wiederum von einer Hülle umgeben ist, die aus dem Oberflächenprotein HBsAg (engl. „surface antigen“) aufgebaut ist [14].

Eine Infektion mit HBV kann sehr unterschiedlich verlaufen. Während sie bei etwa einem Drittel der Erwachsenen asymptomatisch verläuft, kommt es bei bis zu 10 % der Patienten mit akuter Hepatitis zu einer Progression zum chronischen Verlauf. Im Laufe einer chronischen HBV-Infektion besteht die Gefahr der Entwicklung einer Leberzirrhose oder eines Leberzellkarzinoms. Die Infektions- bzw. Krankheitsstadien können anhand der Kombination von HBV-Genom, HBV-Antigenen und Antikörpern gegen HBV-Proteine nachgewiesen werden. Die Inkubationszeit von der HBV-Infektion bis zur Etablierung von klinischen Symptomen beträgt durchschnittlich 60 bis 120 Tage. Für die Transfusionsmedizin ist jedoch bereits die Phase vor Einsetzen der klinischen Symptome ausschlaggebend, wenn sich das Virus asymptomatisch vermehrt.

**Figure 1:**
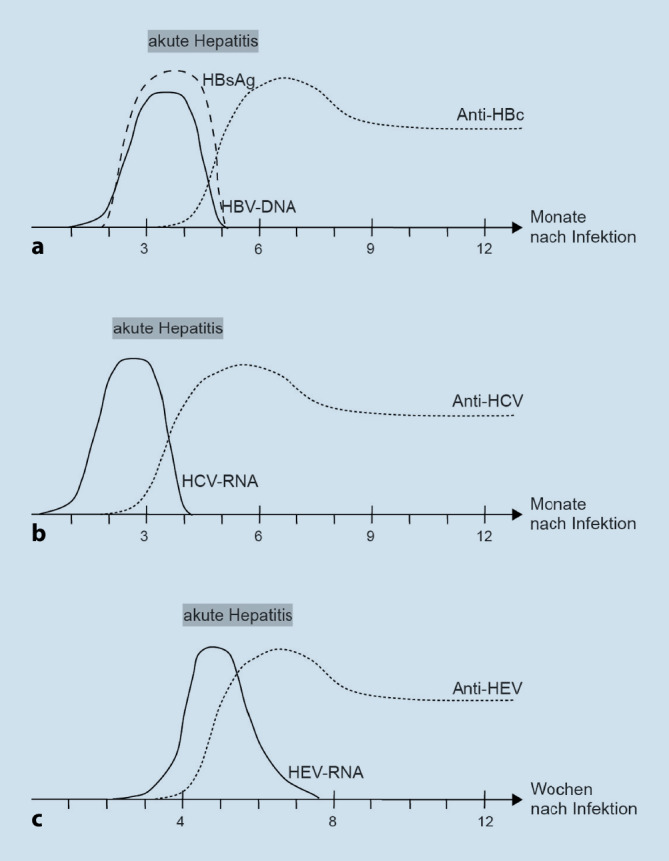

Eine HBV-Infektion lässt sich meist erst nach einigen Wochen durch den Nachweis von HBV-DNA detektieren, gefolgt vom HBsAg-Nachweis weitere Wochen später, bevor schließlich noch weitere Wochen später auch Antikörper gegen HBc (Anti-HBc) gebildet und detektiert werden können (Abb. 1). Bei asymptomatischer Infektion und einer ausheilenden akuten Hepatitis nimmt die Menge an HBV-DNA und HBsAg während des Infektionsverlaufes wieder ab, während bei einer chronischen Infektion HBV-DNA und HBsAg oft über Monate und Jahre nachweisbar sind. Anti-HBc-Antikörper sind im Allgemeinen jahre- bis lebenslang im Körper nachweisbar [14, 15].

Aktuell ist für Spenderblut in Deutschland die Testung auf HBsAg und Anti-HBc vorgeschrieben (siehe oben). Um die sogenannte diagnostische Fensterperiode – die Zeit, in der das Virus im Blut vorhanden ist, aber noch nicht nachgewiesen werden kann, – zu verringern, testen die meisten Blutspendeeinrichtungen zusätzlich freiwillig auf HBV-DNA. Die diagnostische Fensterperiode kann somit um durchschnittlich 2,1–4,9 Tage im Vergleich zur HBsAg-Testung reduziert werden (siehe Abschnitt zur HBsAg-Testung).

### HCV

HCV ist ebenfalls weltweit verbreitet mit geschätzten 71 Mio. chronisch Infizierten und 399.000 Todesfällen im Jahr 2015 [1]. Die Anzahl der 2019 in Deutschland gemeldeten HCV-Erstdiagnosen betrug 5940 Fälle. Das Virus wird fast ausschließlich durch den Kontakt mit kontaminiertem Blut übertragen. Während in Deutschland der unsichere injizierende Drogenkonsum den häufigsten Übertragungsweg darstellt, sind weltweit zusätzlich nosokomiale Übertragungen für einen großen Teil der Infektionen verantwortlich, die in Deutschland keine Rolle spielen [13]. HCV ist ein Hepacivirus aus der Familie der *Flaviviridae* mit einem einzelsträngigen RNA-Genom positiver Polarität, welches von einem Kapsid und einer Virushülle umgeben ist [16].

Etwa 75 % der HCV-Infizierten bleiben symptomfrei, der Rest entwickelt nach einer Inkubationszeit von ca. 40 bis 120 Tagen eine akute, aber meist recht milde Hepatitis. Die Mehrzahl der akuten Hepatitiden persistiert allerdings in Form einer chronischen Hepatitis jahrelang im Körper. Als Spätfolgen entwickeln etwa 16–20 % der Patienten mit chronischer Hepatitis C nach 20 Jahren eine Leberzirrhose, was wiederum mit einem erhöhten Risiko für das Leberzellkarzinom einhergeht. HCV-Infektionen werden beim Spenderscreening über den Nachweis von HCV-RNA und Anti-HCV-Antikörpern detektiert. Eine Serokonversion findet 45–51 Tage nach HCV-Infektion statt [17], gebildete Antikörper bleiben daraufhin jedoch unabhängig vom klinischen Krankheitsbild meist lebenslang nachweisbar. HCV-RNA ist im Gegensatz dazu bereits deutlich früher nachweisbar, nimmt jedoch im Laufe einer ausheilenden akuten HCV-Hepatitis wieder ab (Abb. 1). Bei chronischen Verläufen ist das HCV-Genom meist durchgehend nachweisbar [15, 16].

### HEV

HEV ist ein kleines, unbehülltes Virus der Familie der *Hepeviridae* mit einem einzelsträngigen, positiv-polarisierten RNA-Genom. HEV wurde ursprünglich eher als ein Problem in Entwicklungsländern betrachtet, das hauptsächlich als reiseassoziierte Krankheit in Industrienationen eingeschleppt wird. In den letzten 10 Jahren hat HEV jedoch weltweit an Bedeutung gewonnen mit geschätzten 20 Mio. HEV-Infektionen jährlich [1]. Hepatitis E und ihr Erreger HEV weisen je nach Region und Genotyp deutliche Unterschiede in Epidemiologie und Klinik auf. In vielen Ländern Asiens und Afrikas kommen hauptsächlich die HEV-Genotypen 1 und 2 endemisch vor. Übertragung erfolgt hier aufgrund unzureichender Trinkwasser- und Lebensmittelhygiene meist durch mit menschlichen Fäkalien kontaminiertes Trinkwasser. Für die HEV-Genotypen 1 und 2 ist der Mensch das einzige bekannte Reservoir.

In Deutschland sowie anderen Industrienationen Europas und Nordamerikas ist vor allem der HEV-Genotyp 3 (HEV-3) endemisch verbreitet. Die Übertragung erfolgt hauptsächlich zoonotisch über den Verzehr von unzureichend gegartem Fleisch und daraus hergestellten Produkten. Das Hausschwein ist hierbei das vermutlich wichtigste tierische Reservoir. Das Virus kann auch parenteral (z. B. durch kontaminierte Blutprodukte) übertragen werden. Mensch-zu-Mensch-Übertragungen von Infektionen mit HEV‑3 im Alltag sind jedoch extrem selten. Die Zahl der in Deutschland gemeldeten Fälle steigt seit Jahren kontinuierlich an und lag 2019 bei 3724, was aber auch auf die allgemein vermehrte Aufmerksamkeit für HEV in der Wissenschaft und Medizin zurückzuführen sein kann [2]. Eine Studie von 2014 zeigte eine Seroprävalenz von 6,8 % unter deutschen Blutspendern [18]. Aufgrund des vermehrten Auftretens transfusionsbedingter HEV-Übertragungen [4, 19, 20] wurde das Screening von HEV-RNA in Blutspenden mittlerweile in Deutschland und vielen anderen europäischen Ländern verpflichtend angeordnet (siehe oben).

Eine HEV-Infektion verläuft in der Mehrzahl der Fälle asymptomatisch, führt jedoch nach einer Inkubationszeit von ca. 2–6 Wochen in etwa 5–20 % zu einer akuten Hepatitis, welche in der Regel selbstlimitierend wieder ausheilt. Eine chronische Verlaufsform wurde bisher meist nur in immunsupprimierten Patienten diagnostiziert. HEV-RNA im Blut ist kurz vor Beginn der Symptome gegen Ende der Inkubationszeit detektierbar, kann jedoch etwa 2–4 Wochen nach Krankheitsbeginn nicht mehr nachgewiesen werden. Serokonversion findet kurz nach akutem Krankheitsbeginn statt und führt zu einem meist persistierenden Anti-HEV-Antikörpertiter (Abb. 1; [3, 21]).

## Nachweis erregerspezifischer Nukleinsäuren

Erregerspezifische Nukleinsäuren sind bei den meisten blutübertragenen Viren der erste im Blut nachweisbare Infektionsmarker (siehe oben). NAT-Methoden ermöglichen es somit, Hepatitisvirusinfektionen früh zu erkennen und die diagnostische Fensterperiode, in der das Virus zwar bereits im Körper vorhanden, aber noch nicht nachweisbar ist, möglichst gering zu halten. Zudem lässt die Anwesenheit von Virusgenom direkt auf eine hohe Ansteckungsgefahr schließen.

**Figure 2:**
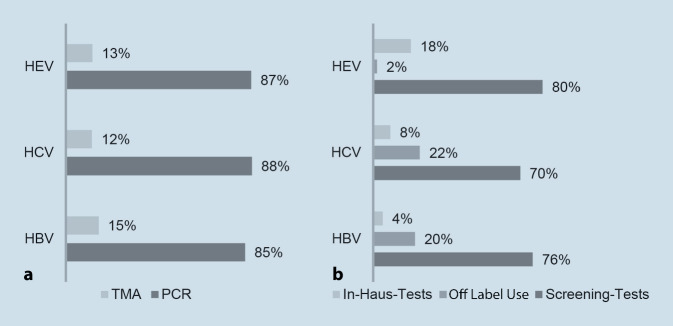

Zum Nachweis von erregerspezifischen Nukleinsäuren werden beim Spenderscreening 2 verschiedene Techniken eingesetzt. Die überwiegende Anzahl (85–88 %) deutscher Blutspendeeinrichtungen verwendet die (Echtzeit‑)Polymerasekettenreaktion (PCR) zum Nachweis von Hepatitisviren in Spenderblut, während die *Transcription-mediated Amplification* (TMA) in 12–15 % der deutschen Blutspendeeinrichtungen zum Einsatz kommt (Abb. 2). Andere NAT-Techniken, wie z. B. LAMP (*Loop-mediated Isothermal Amplification*), werden aktuell nicht angewendet. Mithilfe von PCR und TMA können bereits geringe Mengen an Erregergenom vervielfältigt, detektiert und somit nachgewiesen werden.

Aktuell wird für das Spenderscreening in Deutschland eine Mindestnachweisgrenze von 5000 Internationalen Einheiten (IE) HCV-RNA pro mL bzw. 2000 IE/mL HEV-RNA bezogen auf die Einzelspende gefordert [11, 12]. In Deutschland ist eine Testung mit einer Poolgröße von bis zu 96 Einzelspenden gestattet – vorausgesetzt die Sensitivität der verwendeten Tests entspricht den genannten Anforderungen und entsprechende Validierungen wurden vorgenommen. Aufgrund des rasanten, technischen Fortschritts im Bereich der In-vitro-Diagnostika (IVD) wurden in den letzten 10 Jahren mehrere hochsensitive, automatisierte Systeme in den europäischen Markt eingeführt, welche bereits geringe Mengen an Erregergenom detektieren können. Die meisten heutzutage in Deutschland verwendeten NAT-Screeningsysteme (Tab. 2) greifen auf automatisierte Plattformen zurück, welche die Extraktion der Nukleinsäure mit der Detektion des Erregers in einem Gerät vereinen. Somit sind aktuell analytische Sensitivitätsgrenzen (95 % Limit of Detection, LoD) von bis zu 1,4 IE HBV-DNA, 3,0 IE HCV-RNA bzw. 7,9 IE HEV-RNA pro mL bezogen auf die Einzelspende möglich.

Da die PCR auf der Vervielfältigung von DNA beruht, wird für den Nachweis von HCV- und HEV-Erregern das RNA-Genom in einem vorherigen Schritt durch das Enzym Reverse Transkriptase in komplementäre DNA (cDNA) umgeschrieben, bevor es zu einer zyklischen, exponentiellen Vermehrung von kurzen, gezielten Genabschnitten der Hepatitiserreger kommt. Durch ein spezifisches Primerdesign in konservierten Bereichen des Genoms ist oft die Amplifizierung eines Genomabschnittes ausreichend. Die Detektion und Quantifizierung erfolgt anhand der Echtzeit-PCR-Technologie (Real-Time-PCR) durch fluoreszenzmarkierte Sonden, wobei die freigesetzte Fluoreszenzintensität proportional zur amplifizierten Genommenge ist [22].

Die TMA ist im Gegensatz zur PCR eine isotherme Methode und vervielfältigt Genomabschnitte mithilfe der beiden Enzyme RNA-Polymerase und Reverse Transkriptase. Die Reverse Transkriptase schreibt mithilfe erregerspezifischer Primer die RNA-Zielsequenz in eine DNA-Kopie um, die daraufhin der RNA-Polymerase als Vorlage zur Produktion mehrerer RNA-Kopien dient, welche wiederum von der Reversen Transkriptase in DNA-Kopien umgeschrieben werden. Die entstandenen RNA-Kopien können nun mit einer erregerspezifischen, markierten Sonde detektiert werden [23].

Eine Studie des Deutschen Roten Kreuzes zeigte bereits 1999 den Nutzen und die Machbarkeit einer systematischen NAT-Testung mittels In-Haus-Methoden und kommerzieller PCR auf [24]. Nach der Einführung der verpflichtenden NAT-Testung ist auch der europäische Markt für kommerzielle IVD stetig gewachsen. Während vor 20 Jahren noch die Mehrzahl der Laboratorien eigens etablierte In-Haus-Methoden zum Blutspenderscreening verwendete, so greifen aktuell 70–80 % der Blutspendeeinrichtungen in Deutschland auf kommerzielle IVD zurück, die speziell für das Spenderscreening validiert und zugelassen wurden (Abb. 2). Die restlichen Einrichtungen verwenden entweder In-Haus-Methoden oder kommerzielle IVD im Off Label Use. Sowohl bei Verwendung von IVD im Off Label Use als auch bei In-Haus-Methoden müssen die Blutspendeeinrichtungen jedoch zusätzliche Validierungsmaßnahmen vornehmen, bevor das PEI die Verwendung dieser Tests zum Spenderscreening genehmigt. Aufgrund der erst kürzlich geänderten Vorschriften zur HEV-Testung und einer geringeren Anzahl an kommerziellen HEV-NAT-Systemen auf dem europäischen Markt ist der Anteil der Einrichtungen, die HEV In-Haus-Tests durchführen, mit 18 % noch deutlich höher im Vergleich zur HBV- und HCV-Testung (Abb. 2).

## Nachweis von Antikörpern gegen HCV

Serologische Assays zum Nachweis von Antikörpern gegen HCV (Anti-HCV) wurden nach Entdeckung des Virus im Jahr 1989 entwickelt und seitdem kontinuierlich verbessert. Die heute verwendeten Anti-HCV-Assays der 3. Generation enthalten rekombinante Proteine oder Peptide des HCV-Kernproteins (Core) und der nichtstrukturellen Proteine NS3, meist noch NS4 sowie in einigen Tests zusätzlich NS5. Die Sensitivitätsverbesserung gegenüber der 2. Generation ist in erster Linie auf Rekonfiguration von NS3 durch Reduktionsmittel wie Dithiothreitol (DTT) oder Cystein zurückzuführen, wodurch HCV-Verläufe, die überwiegend durch Anti-NS3 bestimmt sind, früher erkannt bzw. nicht mehr verpasst werden. Eine ausgewogene Sensitivität gegenüber den immunogenen Hauptantigenen Core und NS3 ist daher nach wie vor ein wichtiger Bestandteil bei der Bewertung von neuen Anti-HCV-Screeningtests [11, 25]. Eine weitere Verbesserung der Sensitivität wurde in den letzten Jahren durch „Sandwich“-Tests (anstelle von indirekten IgG-Tests) erreicht, die Anti-HCV-Gesamtantikörper nachweisen, wodurch HCV-Verläufe mit Anti-HCV-IgM früher erkannt werden können.

**Figure 3:**
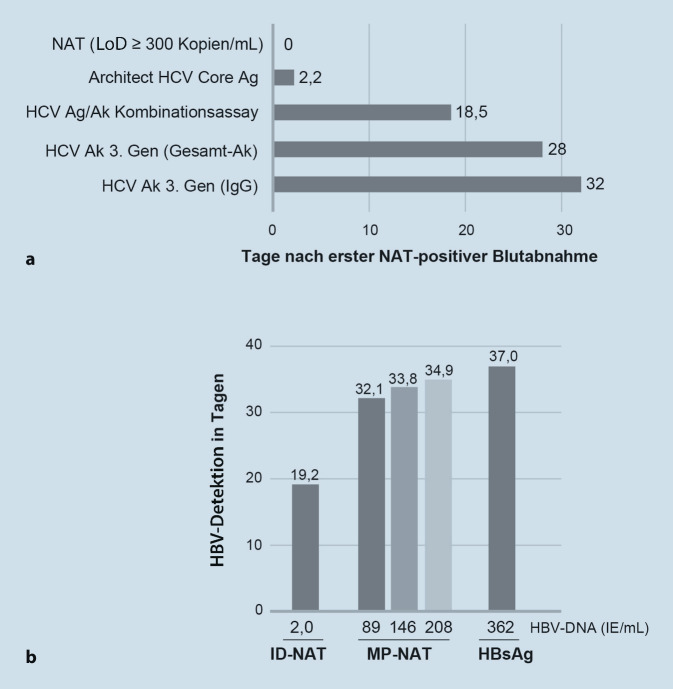

Basierend auf Daten des PEI-IVD-Prüflabors in 17 kommerziell erhältlichen HCV-Serokonversionspanels (Abb. 3a) beträgt die diagnostische Fensterperiode für Anti-HCV-IgG-Screeningtests durchschnittlich 32 Tage (14–42) bzw. ca. 28 Tage mit Anti-HCV-Sandwichtests (Gesamtantikörper) bezogen auf eine HCV-NAT mit einer Nachweisgrenze von 10^3^ Virus-RNA-Kopien/mL. Darüber hinaus sind kombinierte HCV-Antigen/Antikörper-Tests verfügbar, die gleichzeitig sowohl HCV-Core-Antigen als auch HCV-Antikörper nachweisen. Diese Assays können die diagnostische Fensterperiode für den serologischen HCV-Nachweis auf Median 18,5 Tage verkürzen im Vergleich zur HCV-PCR (Abb. 3a), werden aber offenbar bisher wenig eingesetzt verglichen z. B. mit den weitverbreiteten HIV-Ag/Ak-Assays der 4. Generation. Abb. 3a zeigt auch die diagnostische Fensterperiode für einen reinen HCV-Antigentest, der diese im Vergleich zur PCR auf 2,2 Tage deutlich verkürzt. Dieser Test wird jedoch nicht im Blutspenderscreening eingesetzt.

Die Genotypdiversität von HCV zeigte keinen Einfluss auf die Sensitivität der Anti-HCV-Assays [26]. Offenbar scheint die Kombination der Antigenregionen von Core, NS3 und NS4, mit denen die Anti-HCV-Tests beschichtet sind, ausreichend konserviert zu sein, um alle 6 HCV-Genotypen zuverlässig erfassen zu können.

## Nachweis des Hepatitis-B-Oberflächenantigens

Der Nachweis des Hepatitis-B-Oberflächenantigens basiert auf dem Nachweis der „a“-Determinante des HBV-S-Gens, das bei allen HBV-Isolaten vorkommt. Als Testprinzip wird in der Regel ein Sandwichtestdesign verwendet, bei dem das HBs-Antigen durch 2 verschiedene spezifische Antikörper von 2 Seiten zwischen Fest- und Konjugatphase gebunden wird. Die Sensitivität ist an einem internationalen Standard angeglichen und wird in Internationalen Einheiten pro mL (IE/mL) angegeben [27]. Die CE-gekennzeichneten Tests variieren derzeit zum Teil relativ stark in einem weiten Bereich von 0,13–0,006 IE/mL. Der Stufenplan des PEI zur Bewertung von Tests im Spenderscreening [11] soll sicherstellen, dass nur sensitive HBsAg-Tests im Blutspenderscreening eingesetzt werden.

Abb. 3b zeigt das Timing des HBV-Nachweises in der anti-HBc-negativen diagnostischen Fensterperiode einer akuten Hepatitis-B-Infektion. HBsAg wird durchschnittlich 37 Tage nach Infektion (bei einer infektiösen HBV-Dosis von 1 HBV-DNA-Kopie) nachgewiesen, gegenüber 32,1 bis 34,9 Tagen bei Minipool-(MP-)NAT, die überwiegend in Deutschland verwendet wird (LoD 89 bis 208 HBV-DNA-IE/mL bezogen auf die Einzelspende), und 19,2 Tagen bei Einzelspenden (ID-)NAT (LoD ≥ 2 IE/mL; [28]). Die Bewertung des Zeitpunkts des HBV-Nachweises mit den verschiedenen HBV-Tests (HBsAg, MP- und ID-NAT) ermöglicht einen Vergleich der Tests im Hinblick auf ihre Fähigkeit, das HBV-Übertragungsrisiko in der anti-HBc-negativen HBV-Fensterperiode zu verringern. Dieses war bei HBsAg mit 1:1.270.000 bis 1:1.620.000 bereits relativ gering und konnte durch MP-NAT (LoD 146 IE/mL) um den Faktor 1,7 bzw. ID-NAT um den Faktor 3,8 weiter reduziert werden. Es wird außerdem darauf hingewiesen, dass es neuere HBsAg-Tests gibt (Lumipulse G HBsAg-Quant, Architect HBsAg Next Qualitative, Alinity i HBsAg Next Qualitative; [29, 30]), die eine 4‑ bis 5‑fach höhere Sensitivität aufweisen als die derzeit verwendeten HBsAg-Tests und damit in einem Sensitivitätsbereich liegen, der mit der Sensitivität von HBV-DNA-Minipool-Testung vergleichbar ist [28].

Ein wichtiger weiterer Gesichtspunkt sind HBV-Genotypen und HBsAg-Mutanten, die die Sensitivität von HBsAg-Tests stark reduzieren können [28, 31–33]. In dieser Hinsicht erwies sich ein Testdesign als robust, bei dem mehrere Antikörper verwendet werden, um möglichst viele Epitope der HBsAg-„a“-Determinante abzudecken, z. B. 1 oder 2 monoklonale Antikörper auf der Festphase und mehrere monoklonale oder polyklonale Antikörper auf der Konjugatphase (Multiple/Poly- oder Mono/Poly-Design; [33]), obwohl in seltenen Fällen HBsAg-Mutanten weiterhin weniger gut nachgewiesen werden konnten [28].

## Nachweis des Hepatitis-B-Core-Antigens

Während HBsAg und HBV-DNA nach der akuten Phase verschwinden können, bleiben Antikörper gegen das Hepatitis-B-Core-Antigen (Anti-HBc) in der Regel viele Jahre, oft lebenslang, erhalten. Anti-HBc kann der einzige nachweisbare Marker einer abgeklungenen HBV-Infektion sein, wenn Anti-HBs auf nicht nachweisbare Werte zurückgeht und HBsAg sowie HBV-DNA nicht nachweisbar sind. Anti-HBc eignet sich daher zum Nachweis von potenziell infektiösen HBV-Spenden bei okkulter HBV-Infektion. Die derzeit verfügbaren CE-gekennzeichneten Anti-HBc-Tests sind relativ einheitlich hochsensitiv [34], haben eine Spezifität von mehr als 99,5 % bei Blutspendern [35] und liegen damit im gleichen Spezifitätsbereich wie bei anderen Screeningmarkern.

Tab. 3 zeigt die Ergebnisse zu Anti-HBc bei 10.000 Anti-HBc-vorgescreenten Blutspenden. Es gab 188 Anti-HBc-reaktive Proben, die auf zusätzliche HBV-Marker und in 9 Anti-HBc-Tests detailliert analysiert wurden [35]: 89,4 % waren zusätzlich positiv für Anti-HBs, 59,6 % positiv für Anti-HBe, alle waren HBsAg- und HBeAg-negativ, eine Probe war HBV-DNA-positiv, 6,9 % waren isoliert Anti-HBc-positiv („Anti-HBc only“; [36]); 86,2 % der 188 Anti-HBc-positiven Proben waren übereinstimmend in allen 9 Anti-HBc-Tests positiv, 10,1 % in ≥ 5 und 3,7 % in < 5 Anti-HBc-Tests. Diese Studien ergaben somit, dass die überwiegende Mehrheit der Anti-HBc-reaktiven Proben als echt positiv einzustufen war.

**Table 3:** 

			Anti-HBc-Reaktivität in 9 verschiedenen Anti-HBc-Tests		
			Klasse		
Gruppe	Reaktivität in zusätzlichen HBV-Markern	*N* Total (188)	A) 9 Tests	B) 5–8 Tests	C) < 5 Tests
1)	Anti-HBc only	13 (6,9 %)	0	8	5
2)	Anti-HBc + Anti-HBs	62 (33,5 %)	50	11	2
	Anti-HBc + Anti-HBe	7 (3,7 %)	7	0	0
3)	Anti-HBc + Anti-HBs + Anti-HBe	105 (55,9 %)	105	0	0
	Anti-HBc + Anti-HBs + HBV-DNA	1 (0,53 %)	1	0	0
–	–	–	162 (86,2 %)	19 (10,1 %)	7 (3,7 %)
–	–	(168 (89,4 %) insgesamt Anti-HBs positiv)			
		(112 (59,6 %) insgesamt Anti-HBe positiv)			

Daten aus [35]

Gruppe nach Anzahl der detektierbaren HBV-Marker: 1) isoliert Anti-HBc reaktiv („Anti-HBc only“); 2) positiv auf einen zweiten HBV-Marker (Anti-HBs oder Anti-HBe); 3) positiv auf 3 HBV-Marker (Anti-HBc plus Anti-HBs, plus Anti-HBe oder HBV-DNA)

Klasse nach Häufigkeit von Anti-HBc-positiv in allen 9 Anti-HBc-Tests: A) reaktiv in allen 9 Anti-HBc-Tests, B) reaktiv bei 5–8 Anti-HBc-Tests; C) reaktiv bei 4 oder weniger Anti-HBc-Tests

Nach Abklärung der Spezifität der Anti-HBc-Tests und weiterführenden Diskussionen, wonach von 1995 bis 2003 insgesamt 18 gesicherte HBV-Übertragungen ans PEI gemeldet wurden, wovon 7 durch Anti-HBc-Testung zu verhindern gewesen wären, folgte ein Beschluss des Arbeitskreises Blut [37]. Dies führte 2006 schließlich zur Einführung der Spendertestung auf Antikörper gegen Hepatitis-B-Core-Antigen [10] und zum aktuellen Testalgorithmus des Arbeitskreises Blut [38] für die Bestätigung des HBV-Status durch eine hochsensitive HBV-DNA-NAT (≤ 5 IE/mL) oder mindestens einen zusätzlichen Anti-HBc-Test in einem anderen Testformat. Dieser Anti-HBc-Abklärungsalgorithmus wurde in Blutspendeeinrichtungen im Wesentlichen bestätigt [39] mit nur geringfügigen Unterschieden und ohne, dass dabei die Virussicherheit beeinträchtigt war.

## Erfolg der regulatorischen Maßnahmen

Sowohl jeder begründete Verdacht als auch der Nachweis einer transfusionsbedingen Infektion sind meldepflichtig (gemäß §19 Abs. 1 TFG) und unverzüglich dem PEI anzuzeigen. Dieses etablierte Meldesystem trägt dazu bei, den Erfolg eingeführter Maßnahmen und somit die Sicherheit von in Deutschland verwendeten Blutprodukten systematisch und stetig zu bewerten.

Die verpflichtende Einführung der HCV-NAT-Testung von Blutspenden im Jahr 1999 zeigte einen direkten und deutlichen Erfolg für die Sicherheit von Blut und Blutprodukten. Während von 1997 bis 1998 noch insgesamt 19 transfusionsbedingte HCV-Infektionen registriert wurden, beschränkt sich die Anzahl der berichteten Übertragungen in den folgenden 20 Jahren (von 1999 bis 2019) auf 3 nachgewiesene Fälle [40–42]. Die 3 Übertragungsfälle gehen auf 2 Blutspenden während der frühen diagnostischen Fensterperiode der Infektion zurück, wobei die HCV-Konzentrationen im Blut zu gering waren, um sie mit der verwendeten NAT-Methode zu detektieren [43, 44]. Zusätzlich wurden im Zeitraum von 1999 bis 2015 in Deutschland 153 Blutproben als HCV-positiv nachgewiesen, die ohne HCV-NAT nicht identifiziert worden wären [40, 41], was wiederum die Wichtigkeit der HCV-NAT-Testung unterstreicht.

Die Einführung der Anti-HBc-Testung im Jahr 2006 zusammen mit der freiwilligen Etablierung der HBV-NAT zur etwa gleichen Zeit in den meisten Blutspendeeinrichtungen hatte einen ähnlich positiven Effekt auf die Anzahl der Virusübertragungen wie oben für HCV beschrieben. Von 1997 bis 2006 wurden durchschnittlich 2 transfusionsbedingte HBV-Infektionen pro Jahr in Deutschland nachgewiesen [41]. Seit 2007 wurde die Zahl auf insgesamt noch 5 HBV-Übertragungsfälle reduziert (Daten verfügbar bis einschließlich 2019; [28, 42, 45]). Aufgrund der simultanen Einführung der Anti-HBc-Testung zusammen mit der HBV-NAT-Testung in mittlerweile fast allen deutschen Blutspendeeinrichtungen kann der Rückgang der transfusionsbedingen HBV-Übertragungsfälle nicht eindeutig auf eine der beiden Nachweismethoden zurückgeführt werden. Unter den 2019 im Rückverfolgungsverfahren bestätigten HBV-positiven Blutspenden befanden sich Fälle, die im initialen Screening isoliert positiv für jeweils nur einen der 3 Marker (HBsAg, Anti-HBc, HBV-NAT) getestet wurden [42]. Angesichts der geringen HBV-Übertragungsfälle der letzten Jahre und somit einer hohen Sicherheitslage besteht aktuell kein definierbar hohes Risiko, welches eine Änderung des gültigen Testalgorithmus bedingt.

Ob die Verpflichtung zur HEV-NAT seit 2020 einen vergleichbaren Erfolg für die Sicherheit von Blutprodukten erzielt, wird sich in den nächsten Jahren zeigen. Eine Modellierung von 2017 basierend auf Daten zur HEV-Prävalenz bei Blutspendern in Deutschland zeigte auf, dass die Einführung einer HEV-NAT mit einer angenommenen Poolgröße von 96 und Sensitivität von 20 IE/mL 80 % der erwarteten HEV-Übertragungen und die daraus resultierenden chronischen Infektionen verhindern würde [46]. Seit 2013, dem Beginn der Aufzeichnung von transfusionsbedingten HEV-Infektionen, wurden dem PEI bis einschließlich 2019 23 HEV-Übertragungen gemeldet – 10 davon allein 2019 [42]. Die Dunkelziffer kann hier aber durchaus noch höher liegen, da die Aufmerksamkeit für HEV-Infektionen erst in den letzten Jahren stetig angestiegen ist. Im Jahr 2020, dem ersten Jahr mit verpflichtender HEV-Testung, konnte bereits ein erster Effekt der Maßnahme auf die PEI-Meldedaten beobachtet werden. So hat sich die Anzahl der eingeleiteten Rückverfolgungsverfahren für HEV von 947 im Jahr 2019 auf 2771 im Jahr 2020 fast verdreifacht. Gleichzeitig wurde 2020 nur eine transfusionsbedingte HEV-Übertragung gemeldet [47]. Ob dieser Trend auch in den nächsten Jahren anhält, wird weiter beobachtet werden.

## Fazit

Seit den vermehrten Virusübertragungsfällen der 1980er- und 1990er-Jahre konnte die Sicherheit von Blut und Blutprodukten durch Einführung neuer Testanforderungen und deren kontinuierliche Anpassung fortlaufend verbessert und dem Stand der Technik angepasst werden. Mithilfe der etablierten serologischen und NAT-basierten Nachweismethoden bei Blutspenden konnte somit die Anzahl an transfusionsbedingten HCV- und HBV-Infektionen in den letzten 20 Jahren auf ein Minimum mit wenigen Fällen reduziert werden. Mit Einführung der HEV-Spendertestung im Jahr 2020 ist die Wahrscheinlichkeit hoch, auch die gemeldeten HEV-Übertragungen auf seltene Einzelfälle zu minimieren. Aufgrund der zugrunde liegenden Regulationsstruktur ist es auch in Zukunft möglich, die Testalgorithmen bei Bedarf an neue Datenlagen anzupassen, um die Sicherheit von Blutspenden weiterhin zu gewährleisten. Bei jährlich etwa 4 Mio. Blutspenden in Deutschland kann die Sicherheit von Blutprodukten insgesamt als sehr hoch und das Risiko einer Übertragung mit Hepatitisviren als sehr gering bewertet werden.
